# Supplementary material for: Epidemiology of Mycobacterium tuberculosis lineages and strain clustering within urban and peri-urban settings in Ethiopia
Source: PLoS One. 2021 Jul 12;16(7):e0253480. doi: 10.1371/journal.pone.0253480 (PMC8274931; doi:10.1371/journal.pone.0253480)
Supplement: S1 File — (DOCX) [file pone.0253480.s002.docx]

**Major steps and software codes**

Except the Multi-level (hierarchical logistic regression) model which is performed using STATA software, all other statistical analysis was done under R-studio using different commands under "epicalc" software package. Before the executing the final analysis, we have gone through the following steps

- library(epicalc)
- attach(SPO)
- use(SPO)
- des()
- labelling and factoring variables such as
  - Clustering <- factor(Clustering, labels = c('Unique','Clustered'))
  - Orphan <- factor(Orphan, labels = c('Identified','New or Orphan'))
  - SNP <- factor(SNP, labels = c('L3','L4','Others'))
  - KBBN<- factor(KBBN, labels = c('CAS','Haarlem', 'LAM','Manu','T','T3','T3-ETH','Others'))
  - CBN <- factor(CBN, labels = c('L4','L3'))
- Collapsing level of category (when necessary)
- Reference leveling such as
  - CBN = relevel(factor(CBN), ref = "L3")
  - Diagnosis = relevel(factor(Diagnosis), ref = "PTB")
  - HIV = relevel(factor(HIV), ref = "No")
  - Region = relevel(factor(Region), ref = "Gondar")

1. **Table 4 (Multivariable analysis)**

- Model selection (backward variable exclusion) was performed using "Step" command under R-studio
- $Table 4=$step(glm(Clustering ~ Region+Gender+Diagnosis+BCG+…..+SNP ,data = SPO),direction = "backward")

Then the final model that includes clinical relevant variables selected from the Model with lowest AIC Value:

- $\mathrm{GLM} =$glm(Clustering ~ Region + Diagnosis + HIV + CCD + Bcough + SNP, family = binomial)

The output (OR and 95% CI and the P-value was generated using the following command

- $Table 4= logistic.display(\mathrm{GLM},crude.p.value = TRUE$ )

Whereas the Multi-level (hierarchical logistic regression) was generated from STATA software using the following "melogit" command

- melogit Clustering i.Diagnosis i.HIV i.CCD i.Bcough i.SNP || Region: ,or

1. **Table 4 (Multivariable analysis)**

After excluding seven observations (cases infected with other lineages), the clinical characteristics of 251 participants, who were caused by (92 L3 and 159 L4) were included in Table 5. Except change in outcome variables and respective independent variables, we followed the same step like that of Table 4.

$GLM2=$glm(CBN ~ Region + Gender+ Diagnosis + HIV + CCD + Pmed2 + Cough + Bcough + Lweight , family = binomial)

- $Table 5= logistic.display(\mathrm{GLM}2,crude.p.value = TRUE$ )
