# Supplementary material for: Epidemiology of Mycobacterium tuberculosis lineages and strain clustering within urban and peri-urban settings in Ethiopia
Source: PLoS One. 2021 Jul 12;16(7):e0253480. doi: 10.1371/journal.pone.0253480 (PMC8274931; doi:10.1371/journal.pone.0253480)
Supplement: Attachment — Submitted filename: PONE-D-20-38732.pdf_Comments by editor..pdf [file pone.0253480.s004.pdf]

# PLOS ONE

## Epidemiology of Mycobacterium tuberculosis and factors associated with strain clustering and lineage predominance around urban and peri-urban settings in Ethiopia.

--Manuscript Draft--


| | |
|---|---|
| **Manuscript Number:** | PONE-D-20-38732 |
| **Article Type:** | Research Article |
| **Full Title:** | Epidemiology of Mycobacterium tuberculosis and factors associated with strain clustering and lineage predominance around urban and peri-urban settings in Ethiopia. |
| **Short Title:** | Spoligotyping based molecular study of Mycobacterium tuberculosis species |


| | |
|---|---|
| **Corresponding Author:** | Hawult Taye Adane, MPH<br>Armauer Hansen Research Institute<br>Addis Ababa, Ethiopia ETHIOPIA |


| | |
|---|---|
| **Keywords:** | Epidemiology; Mycobacterium tuberculosis; spoligotyping; strain clustering; associated factors |


**Abstract:**

Background

The significance of strain diversity in the epidemiology of tuberculosis (TB) has received wider recognition in recent years. Previous work in Ethiopia has shown differential predominance of certain Mycobacterium tuberculosis lineages and sub-lineages among different human populations in diverse geographic regions of the country. Nevertheless, how strain diversity is evolving under the ongoing rapid socio-economic and environmental changes in the region is poorly understood. The present study investigated the diversity of M. tuberculosis strains and factors associated with strain clustering and most dominant lineages within selected urban and peri-urban areas.

Methods

PTB and TBLN patients who visited selected health facilities from four different regions in Ethiopia were recruited in the year 2016 and 2017. A total of 258 M. tuberculosis isolates identified from 163 sputa and 95 fine-needle aspirates were characterized by spoligotyping and the outputs compared with existing spoligotype patterns in the SITVIT2 and linked databases. Molecular data were merged with clinical and demographic data of the patients for further analysis.

Results

From a total of 258 isolates, 84 distinct spoligotype patterns were identified. Of which, 58 SIT patterns (89.5% of all strains) already registered in the SITVIT2 database, while 26 (10.5%) of them were new or orphan patterns. The majority of strains belonged to two major M. tuberculosis lineages, L3 (35.7%) and L4 (61.6%). According to the KBBN classification, 97.8% of L3 strains shared the CAS sub-lineage while the T, T3-ETH and Haarlem sub-lineages made up 67.7% of L4 strains. The observed high percentage of shared patterns (n = 200/258) suggested a substantial rate of overall clustering (77.5%) in the region. After adjusting for the effect of geographical variations, clustering rate was found to be significantly lower among individuals co-infected with HIV and other concomitant chronic disease; with AOR & 95% CI of: 0.16 (0.05, 0.47) and 0.46 (0.23,0.91) respectively. On the other hand, compared to L4, infections with L3 M. tuberculosis strains were more likely to be associated with TBLN [3.47 (1.45, 8.29)] and TB-HIV co-infection [2.84 (1.61, 5.55)]; but inversely associated with other concomitant chronic disease [0.46 (0.25, 0.87)].

Conclusion

Despite the observed difference across regions, strain diversity and distribution of M. tuberculosis lineages was nearly consistent with previous reports in Ethiopia. Nevertheless, an increased rate of strain clustering suggests higher transmission and this should warrant more detailed investigations into the molecular epidemiology of M. tuberculosis and related factors.



| | |
|---|---|
| **Order of Authors:** | Hawult Taye Adane, MPH |
| | Kassahun Alemu |
| | Adane Mihret |
| | |

| | |
|---|---|
| | Sosina Ayalew |
| | Elena Hailu |
| | James L.N Wood |
| | Ziv Shkedy |
| | Stefan Berg |
| | Abraham Aseffa |
| **Opposed Reviewers:** | |
| **Additional Information:** | |
| **Question** | **Response** |
| **Financial Disclosure**<br><br>Enter a financial disclosure statement that describes the sources of funding for the work included in this submission. Review the submission guidelines for detailed requirements. View published research articles from *PLOS ONE* for specific examples.<br><br>This statement is required for submission and **will appear in the published article** if the submission is accepted. Please make sure it is accurate.<br><br>**Unfunded studies**<br>Enter: *The author(s) received no specific funding for this work.*<br><br>**Funded studies**<br>Enter a statement with the following details:<br>• Initials of the authors who received each award<br>• Grant numbers awarded to each author<br>• The full name of each funder<br>• URL of each funder website<br>• Did the sponsors or funders play any role in the study design, data collection and analysis, decision to publish, or preparation of the manuscript?<br>• **NO** - Include this sentence at the end of your statement: *The funders had no role in study design, data collection and analysis, decision to publish, or preparation of the manuscript.*<br>• **YES** - Specify the role(s) played.<br><br>* typeset | This work was funded by the Biotechnology and Biologic Sciences Research Council, the Department for International Development, the Economic & Social Research Council, the Medical Research Council, the Natural Environment Research Council and the Defence Science & Technology Laboratory, under the Zoonoses and Emerging Livestock Systems (ZELS) program, ref: BB/L018977/1. SB was also partly funded by the Department for Environment, Food & Rural Affairs, United Kingdom, ref: TBSE3294. The Armauer Hansen Research Institute is supported by core funds from Norad and Sida. |
| **Competing Interests** | The authors have declared that no competing interests exist. |

| | |
|---|---|
| Use the instructions below to enter a competing interest statement for this submission. On behalf of all authors, disclose any competing interests that could be perceived to bias this work—acknowledging all financial support and any other relevant financial or non-financial competing interests.<br><br>This statement **will appear in the published article** if the submission is accepted. Please make sure it is accurate. View published research articles from *PLOS ONE* for specific examples.<br><br>**NO authors have competing interests**<br><br>Enter: *The authors have declared that no competing interests exist.*<br><br>**Authors with competing interests**<br><br>Enter competing interest details beginning with this statement:<br><br>*I have read the journal's policy and the authors of this manuscript have the following competing interests: [insert competing interests here]*<br><br>* typeset | |
| **Ethics Statement**<br><br>Enter an ethics statement for this submission. This statement is required if the study involved:<br><br>• Human participants<br>• Human specimens or tissue<br>• Vertebrate animals or cephalopods<br>• Vertebrate embryos or tissues<br>• Field research<br><br>Write "N/A" if the submission does not require an ethics statement.<br><br>General guidance is provided below. Consult the submission guidelines for detailed instructions. **Make sure that all** | Ethical clearance was obtained from the AHRI/ALERT Ethics Review Committee and University of Gondar Institutional Review Board (IRB). Support letters were obtained from Regional State Health Bureaus and health facilities. Enrolment of study participants was done after written informed consent was secured and signed agreements were received from all participating health facilities. |

*Powered by Editorial Manager® and ProduXion Manager® from Aries Systems Corporation*

| | |
|---|---|
| **information entered here is included in the Methods section of the manuscript.**<br><br>**Format for specific study types**<br><br>**Human Subject Research (involving human participants and/or tissue)**<br>• Give the name of the institutional review board or ethics committee that approved the study<br>• Include the approval number and/or a statement indicating approval of this research<br>• Indicate the form of consent obtained (written/oral) or the reason that consent was not obtained (e.g. the data were analyzed anonymously)<br><br>**Animal Research (involving vertebrate animals, embryos or tissues)**<br>• Provide the name of the Institutional Animal Care and Use Committee (IACUC) or other relevant ethics board that reviewed the study protocol, and indicate whether they approved this research or granted a formal waiver of ethical approval<br>• Include an approval number if one was obtained<br>• If the study involved *non-human primates*, add *additional details* about animal welfare and steps taken to ameliorate suffering<br>• If anesthesia, euthanasia, or any kind of animal sacrifice is part of the study, include briefly which substances and/or methods were applied<br><br>**Field Research**<br><br>Include the following details if this study involves the collection of plant, animal, or other materials from a natural setting:<br>• Field permit number<br>• Name of the institution or relevant body that granted permission | |
| **Data Availability**<br><br>Authors are required to make all data underlying the findings described fully available, without restriction, and from the time of publication. PLOS allows rare exceptions to address legal and ethical | Yes - all data are fully available without restriction |

| | |
|---|---|
| concerns. See the PLOS Data Policy and FAQ for detailed information.<br><br>A Data Availability Statement describing where the data can be found is required at submission. Your answers to this question constitute the Data Availability Statement and **will be published in the article**, if accepted.<br><br>**Important:** Stating 'data available on request from the author' is not sufficient. If your data are only available upon request, select 'No' for the first question and explain your exceptional situation in the text box.<br><br>Do the authors confirm that all data underlying the findings described in their manuscript are fully available without restriction? | |
| **Describe where the data may be found in full sentences. If you are copying our sample text, replace any instances of XXX with the appropriate details.**<br><br>• If the data are **held or will be held in a public repository**, include URLs, accession numbers or DOIs. If this information will only be available after acceptance, indicate this by ticking the box below. For example: *All XXX files are available from the XXX database (accession number(s) XXX, XXX.*).<br>• If the data are all contained **within the manuscript and/or Supporting Information files**, enter the following: *All relevant data are within the manuscript and its Supporting Information files.*<br>• If neither of these applies but you are able to provide **details of access elsewhere**, with or without limitations, please do so. For example:<br><br>*Data cannot be shared publicly because of [XXX]. Data are available from the XXX Institutional Data Access / Ethics Committee (contact via XXX) for researchers who meet the criteria for access to confidential data.* | All relevant data are within the manuscript and its Supporting Information files. |

| | |
|---|---|
| *The data underlying the results presented in the study are available from (include the name of the third party and contact information or URL).*<br>• This text is appropriate if the data are owned by a third party and authors do not have permission to share the data.<br><br>* typeset | |
| Additional data availability information: | |

Powered by Editorial Manager® and ProduXion Manager® from Aries Systems Corporation

Dear Editor,

It is well known that Tuberculosis (TB) is a major public health issue in many parts of the world but extremely challenging those high burden countries like Ethiopia. The significance of strain diversity in the epidemiology of tuberculosis (TB) has gotten wider recognition in recent years. Previous work in Ethiopia has shown that *Mycobacterium tuberculosis* is the main causative agent in humans and with differential predominance of certain lineages and sub-lineages among different human populations in diverse geographic regions of the country. Some of the strains have a lineage ancestry that dates back longer than others but how strain diversity is evolving under the ongoing rapid socio-economic and environmental changes in the region is poorly understood.

Here we provide you with the research article entitled "**Epidemiology of *Mycobacterium tuberculosis* and factors associated with strain clustering and lineage predominance around urban and peri-urban settings in Ethiopia**". That was aiming to investigate the molecular epidemiology of *Mycobacterium tuberculosis, which* is the main causative agent in humans globally. Using spoligotyping based molecular technique we identified a diverse type of *M. tuberculosis* population structure, dominated by several endemic lineages and sub-lineages circulating in four peri-urban regions of Ethiopia. We had observed substantial strain clustering that suggests higher rate of transmission in the study sites. Using advanced statistical methods, we further investigated the contribution of bacterial genotype and host related factors associated with rate of strain clustering and predominance.

Therefore, we think that the research article was done based on standard scientific procedures and it is well prepared in accordance with the author's guideline and mandatory requirements specified for PLOS ONE.

We hope for a positive review and publication of this study in your Journal.

Best regards

**Corresponding author**
Full Name: Hawult Taye Adane
Email: hawultachew@gmail.com

Manuscript Click here to access/download;Manuscript;Main manuscript.rtf

# Epidemiology of *Mycobacterium tuberculosis* and factors associated with strain clustering and lineage predominance around urban and peri-urban settings in Ethiopia.

Hawult Taye[1,2,*], Kassahun Alemu[2], Adane Mihret[1], Sosina Ayalew[1], Elena Hailu[1], James L.N. Wood[4], Ziv Shkedy[2,3], Stefan Berg[5], Abraham Aseffa[1], The ETHICOBOTS consortium[^]

[*] Corresponding author

Email: hawultachew@gmail.com (HT)

[1] Armauer Hansen Research Institute, Addis Ababa, Ethiopia

[2] Department of Epidemiology and Biostatistics, University of Gondar, Gondar, Ethiopia

[3] Biostatistics and bioinformatics, University of Hasselt, Belgium

[4] Disease Dynamics Unit, Department of Veterinary Medicine, University of Cambridge, Cambridge, United Kingdom

[5] Bacteriology Department, Animal and Plant Health Agency, Weybridge, United Kingdom

[^] Members of the ETHICOBOTS consortium are listed under Acknowledgements

## Abstract

## Background

The significance of strain diversity in the epidemiology of tuberculosis (TB) has received wider recognition in recent years. Previous work in Ethiopia has shown differential predominance of certain *Mycobacterium tuberculosis* lineages and sub-lineages among different human populations in diverse geographic regions of the country. Nevertheless, how strain diversity is evolving under the ongoing rapid socio-economic and environmental changes in the region is poorly understood. The present study investigated the diversity of *M.*

*tuberculosis* strains and factors associated with strain clustering and most dominant lineages within selected urban and peri-urban areas.

## Methods

PTB and TBLN patients who visited selected health facilities from four different regions in Ethiopia were recruited in the year 2016 and 2017. A total of 258 *M. tuberculosis* isolates identified from 163 sputa and 95 fine-needle aspirates were characterized by spoligotyping and the outputs compared with existing spoligotype patterns in the SITVIT2 and linked databases. Molecular data were merged with clinical and demographic data of the patients for further analysis.

## Results

From a total of 258 isolates, 84 distinct spoligotype patterns were identified. Of which, 58 SIT patterns (89.5% of all strains) already registered in the SITVIT2 database, while 26 (10.5%) of them were new or orphan patterns. The majority of strains belonged to two major *M. tuberculosis* lineages, L3 (35.7%) and L4 (61.6%). According to the KBBN classification, 97.8% of L3 strains shared the CAS sub-lineage while the T, T3-ETH and Haarlem sub-lineages made up 67.7% of L4 strains. The observed high percentage of shared patterns (n = 200/258) suggested a substantial rate of overall clustering (77.5%) in the region. After adjusting for the effect of geographical variations, clustering rate was found to be significantly lower among individuals co-infected with HIV and other concomitant chronic disease; with AOR & 95% CI of: 0.16 (0.05, 0.47) and 0.46 (0.23,0.91) respectively. On the other hand, compared to L4, infections with L3 *M. tuberculosis* strains were more likely to be associated with TBLN [3.47 (1.45, 8.29)] and TB-HIV co-infection [2.84 (1.61, 5.55)]; but inversely associated with other concomitant chronic disease [0.46 (0.25, 0.87)].

## Conclusion

Despite the observed difference across regions, strain diversity and distribution of *M. tuberculosis* lineages was nearly consistent with previous reports in Ethiopia. Nevertheless, an increased rate of strain clustering suggests higher transmission and this should warrant more detailed investigations into the molecular epidemiology of *M. tuberculosis* and related factors.

**KEYWORDS**:

Epidemiology; *Mycobacterium tuberculosis;* spoligotyping; strain clustering; associated factors

# Introduction

Tuberculosis (TB) is a chronic infectious disease caused by species of the *Mycobacterium tuberculosis* complex (MTBC). Except for *M. tuberculosis*, which is the primary cause of human TB, other members of the MTBC are believed to have adapted to different animal hosts and therefore they may have reduced fitness to cause human infection [1, 2]. Beside environmental and socio-economic factors, the biology and epidemiology of human TB has likely been shaped by the historical interaction between MTBC members and its host [3].

The genetic variation in MTBC species contributes to the ambiguities concerning disease presentation, frequency of transmission and clinical progress [1, 4]. This is particularly true for *M. tuberculosis*, where the interaction of genotypic variation among different strains with human genetic polymorphism play a prominent

role in the epidemiology of TB diseases [3, 5]. Different alternative molecular identification methods have been used to estimate rates of transmission, which is generally inferred by comparing genotypic clustering between patient isolates from a given epidemiological setting [6, 7]. In other words, successful transmission of particular genotypes has been reflected through an increase in the frequency and consistency of strain domination over time in defined populations [8-10]. Although, there are no well-established classical factors that are known to be strongly associated with disease phenotype, immunological studies reveal that some strains are more virulent and more infectious than others [11].

However; despite recently developed advanced molecular diagnostic tools, both the nature of genotype variations and the characteristics of the host immune response to certain types of *M. tuberculosis* strains are largely unknown in many TB high burden settings [12, 13]. Particularly in countries like Ethiopia, where there is high prevalence and high transmission rate and a diversified population of bacterial species [14-17], molecular identification of the agents can be an important component of the knowledge base required to improve on previous achievements of the national TB control program. Taking all this into account, the present study investigates the molecular epidemiology of TB to identify factors associated with rate of clustering and *M. tuberculosis* lineage predominance, within the context of urban and peri-urban settings in Ethiopia.

# Materials and methods

## Study design and setting

A multi-centre health facility based cross-sectional study was conducted in Ethiopia during 2016 and 2017. As part of the Ethiopia Control of Bovine Tuberculosis Strategies (ETHICOBOTS) project, four hospitals, two private clinics, and fourteen health centers located in urban and peri-urban areas, were purposively selected from four different regions of Ethiopia. Addis Ababa was the largest study site and constituted Addis Ababa city and the surrounding special zone of Oromiya region while the remaining three study sites were located around the regional urban cities of Mekele in Tigray, Gondar in Amhara, and Hawassa in Southern Nations Nationalities, and Peoples' region.

## Study population

Recruitment of participants at selected health facilities was carried out according to the national guideline standard case definition criteria. All presumed TB cases were initially considered as potential source of the study population. Then those patients clinically diagnosed with pulmonary TB (PTB) and cervical TB lymphadenitis (TBLN) were asked for informed consent and enrolled consecutively. Recruitment of PTB cases was done at all selected governmental health facilities. TBLN patients were however recruited from the Pathology Units of three governmental hospitals and private clinics because of lack of diagnostic facilities and skilled professionals for fine-needle aspirate cytology (FNAC) examination at governmental health centers. Included cases from both groups were those eligible for first-line Anti-TB treatment. Known MDR (multi drug resistant) TB cases and extra pulmonary TB (EPTB) patients other than those with TBLN were excluded in this study.

## Data collection

Clinical and demographic information was collected from recruited TB cases using a pre-tested structured questionnaire. Following the routine care service, consented PTB and TBLN participants were requested to provide spot sputum and fine-needle aspirate (FNA) samples, respectively. Care providers (nurses) working at directly observed therapy (DOT) centres were responsible to collect sputum specimens using sterile containers. FNA specimens were collected from the selected hospitals and private clinics by experienced pathologists who performed FNAC examination as part of their routine diagnostic service. According to the standard procedure, FNA collection was performed using a 21-gauge needle attached to a 10 ml syringe and specimens were collected into cryo-tubes with sterile phosphate buffer saline (PBS). Samples were kept at -20°C at remote study sites until transported to the Armauer Hansen Research Institute (AHRI) TB laboratory on dry ice where the clinical samples were stored at -80°C until processed for mycobacterial culture. Clinical sample handling and laboratory procedures were performed according to previously published protocol [15].

## Mycobacterial Culturing

Samples collected in the study were prepared, processed and cultured for mycobacteria using standard procedures established at the AHRI TB laboratory [15, 18]. Specimen samples were inoculated on Löwenstein-Jensen (LJ) medium slants supplemented with either glycerol or pyruvate and incubated at 37°C. The slopes were examined weekly for up to eight weeks for any visible growth. Bacterial colonies identified as Acid-Fast Bacilli by ZN staining [15] were saved as frozen stocks in 20% glycerol as well as heat-inactivated in 500µl distilled $H_2O$ at 80°C for 60 min; the latter samples were used for subsequent molecular identification.

## Molecular identification techniques

All isolates were screened by Large Sequence Polymorphism (LSP) typing using conventional PCR for examination of Region of Difference 9 (RD9) according to protocols by Berg et al. (2009) [19]. Spoligotyping was performed according to Kamerbeek et al. (1997) [20], using a non-commercial biodyne-C-membrane produced by Animal & Plant Health Agency (United Kingdom).

## Genotype analysis and comparison with global databases

Spoligotype patterns were converted into binary and octal formats and compared with previously reported strains in the in-house SITVIT2 proprietary database of Institute Pasteur de la Guadeloupe, which is an updated version of SITVITWEB database [21]. Here, spoligotypes common to more than one strains were designated as shared types (ST) and were assigned a shared international type (SIT) number according to the international spoligotype SITVIT2 database while patterns that did not recognized from updated version of the database were labelled as "New or Orphan". Further lineage classification for corresponding nomenclature was done using 'Run TB-Lineage' online tool; from linked databases (http://www.miru-vntrplus.org/MIRU/index.faces and http://tbinsight.cs.rpi.edu/run_tb_lineage.html). Here, major lineages were predicted using a conformal Bayesian network (CBN) analysis while knowledge based Bayesian network (KBBN) analysis was used to predict the corresponding sub-lineages.

## Data management and Statistical Analysis

All genotype outputs from the computer assisted analyses were imported to SPSS and merged with clinical and demographic data. Once basic data management and cleaning has done the final dataset was exported to STATA and R-software that were preferred to perform further statistical analysis. Two of the main outcome variables were categorized as binomial scale of measurement. In the first logistic regression analysis "clustered"

referred to two or more isolates sharing identical spoligotyping patterns while isolates that did not have shared patterns was defined as “unique”. Here, three different logistic regression analysis methods were performed to identify and compare factors associated with strain clustering. The hierarchical modeling was preferred to adjust for the effect of regional variations where host-related clinical factors and spoligotype-based *M. tuberculosis* lineage classification were considered as second level factors. Thus, an increased rate of *M. tuberculosis* transmission is generally inferred by comparing clustered genotyping patterns of clinical isolates from a given epidemiological setting [6]. By contrast, cases with isolates of a unique pattern could be considered to have resulted from reactivation of latent infection or were else presumably acquired outside of the study population [22]. The second multivariable logistic regression was used to determine the clinical characteristics or disease phenotypes associated with the dominant *M. tuberculosis* lineage. In both cases, stepwise backward elimination technique was applied to minimize the effect of confounding variables. Initially, all clinically relevant factor variables were included in the full model. Then using the specific statistical command (Step) under R-studio, the software program automatically generated all possible alternative models having lists of dependent and independent variables. Finally, according to Likelihood Ratio-test, relatively better fitted model with potential explanatory variables that has the lowest akaki information criteria (AIC) was selected. Independent relationship of variables was decided based on different cut-off point for statistical significance level ($\alpha$: < 0.05; < 0.01 and < 0.001) and interpretation of key findings was reported using adjusted OR (95% CI).

## Ethical considerations

Ethical clearance was obtained from the AHRI/ALERT Ethics Review Committee and University of Gondar Institutional Review Board (IRB). Support letters were obtained from Regional State Health Bureaus and health facilities. Enrolment of study participants was done after written informed consent was secured and signed agreements were received from all participating health facilities.

# Results

## Characteristics of the study population

This study examined a total of 258 TB patients (163 PTB and 95 cervical TBLN) of which 145 (56.2%) were male and 113 (43.8%) were female, with a mean age of 32.2 ± 12.9 years. Most of these TB cases were from Gondar, 111/258 (43.0%), and Mekele, 61/258 (23.6%), in northern Ethiopia while the remaining patients, 44/258 (17.1%) and 42/258 (16.3%), were from Addis Ababa and Hawassa in central and southern Ethiopia, respectively. Farmers (80/258, 31.0%) and students (40/258, 15.5%) were the two most common occupation types in the study population. With regard to the medical history of participants, 20/258 (7.8%) of them were co-infected with HIV and 96/258 (37.2%) had at least one additional chronic concomitant disease. Table 1

**Table 1. Characteristics of the 258 study participants recruited at selected health facilities located in and around urban and peri-urban areas of Ethiopia in the year 2016/17.**

| Characteristics of study participants | Frequency, n (%) |
|---|---|
| **Gender** | |
| Male | 145 (56.2) |
| Female | 113 (43.8) |
| **Occupation** | |
| Farmer | 80 (31.0) |

| | |
|---|---|
| Merchant | 25 (9.7) |
| Employee | 33 (12.8) |
| Student | 40 (15.5) |
| House wife | 37 (14.3) |
| Dairy worker | 16 (6.2) |
| Others | 27 (10.5) |
| **Geographical location** | |
| Gondar | 111 (43.0) |
| Hawassa | 42 (16.3) |
| Mekele | 61 (23.6) |
| Addis Ababa | 44 (17.1) |
| **Clinical diagnosis** | |
| PTB | 163 (63.2) |
| TBLN | 95 (36.8) |
| **HIV co-infection** | |
| No | 238 (92.3) |
| Yes | 20 (7.8) |
| **Chronic concomitant disease** | |
| No | 162 (62.8) |
| Yes | 96 (37.2) |

## Genetic Diversity of Mycobacterium tuberculosis lineages

All isolates were LSP typed as *M. tuberculosis* (intact for RD9). When they were spoligotyped, 84 different patterns were identified, of which 58 SIT patterns accounted for 230 (89.5%) isolates that were already recognized in the SITVIT2 database. Twenty six patterns, representing 27 (10.5%) of the total, were found to be new or orphans; that were not yet recognized in the SITVIT2 database. As presented in Table 2, over half of the isolates 145/258 (56.2%) were represented by five of the dominant SIT patterns, including SIT25 (n = 40), SIT149 (n = 36), SIT53 (n = 32), SIT26 (n = 17), and SIT37 (n = 11). The overall clustering rate aggregated from 26 shared patterns was 77.5% (200/258). Among these, 25 designated shared patterns, each with 2 to 40 isolates, accounted for 86.1% (198/230) of all identified SIT patterns (Table 2) while remaining two shared patterns were new or orphan strains (Table 3).

**Table 2. Spoligotype descriptions of all registered shared SIT patterns that were identified from clinical samples collected from both PTB and TBLN patients recruited at selected health facilities in Ethiopia in the year 2016/17.**

| Spoligotype pattern of shared SIT strains | | | Lineage classification | | | Shared isolates |
|---|---|---|---|---|---|---|
| SIT № | Octal code | Binary format (presence (black) or absence (white) of 43 spacers) | KBBN | CBN | SNP-based Prediction* | |
| 4 | 000000007760771 | □□□□□□□□□□□□□□□□□□□□□□□□■■■■■■■■□□□□■■■■■■■ | T1-RUS2 | EA | L4 | 2 (0.8) |
| 952 | 603777740003771 | ■■□□□□□■■■■■■■■■■■■■■■□□□□□□□□□□□□■■■■■■■■■ | CAS1-Delhi | EAI | L3 | 3 (1.2) |
| 1729 | 700000004177771 | ■■■□□□□□□□□□□□□□□□□□□□□□■□□□□■■■■■■■■■■■■■■ | AFRI | AFRI | L7 | 2 (0.8) |
| 21 | 703377400001771 | ■■■□□□□■■□■■■■■■■■■□□□□□□□□□□□□□□□□■■■■■■■■ | CAS1-Kili | EAI | L3 | 5 (1.9) |
| 2359 | 703677740003171 | ■■■□□□□■■■■□■■■■■■■■■■□□□□□□□□□□□□■■□□■■■■■ | CAS1-Delhi | EAI | L3 | 4 (1.6) |
| 2973 | 703701740003171 | ■■■□□□□■■■■■□□□□□■■■■■□□□□□□□□□□□□■■□□■■■■■ | CAS1-Delhi | EAI | L3 | 2 (0.8) |
| 1199 | 703701740003171 | ■■■□□□□■■■■■■■■■■■■■■■□□□□□□□□□□□□□■□□■■■■■ | CAS1-Delhi | EAI | L3 | 2 (0.8) |
| 25 | 703777740003171 | ■■■□□□□■■■■■■■■■■■■■■■□□□□□□□□□□□□■■□□■■■■■ | CAS1-Delhi | EAI | L3 | 40 (15.5) |
| 26 | 703777740003771 | ■■■□□□□■■■■■■■■■■■■■■■□□□□□□□□□□□□■■■■■■■■■ | CAS1-Delhi | EAI | L3 | 17 (6.6) |
| 1877 | 737377777760771 | ■■■□■■■■■□■■■■■■■■■■■■■■■■■■■■■■□□□□■■■■■■■ | T | EA | L4 | 2 (0.8) |
| 33 | 776177607760771 | ■■■■■■■■□□□■■■■■■■■■□□□□■■■■■■■■□□□□■■■■■■■ | LAM3 | EA | L4 | 3 (1.2) |
| 149 | 777000377760771 | ■■■■■■■■■□□□□□□□□□□■■■■■■■■■■■■■□□□□■■■■■■■ | T3-ETH | EA | L4 | 36 (14.0) |
| 504 | 777737737760771 | ■■■■■■■■■■■■□■■■■■■■■■□■■■■■■■■■■□□□□■■■■■■■ | T3 | EA | L4 | 2 (0.8) |
| 726 | 777737747413771 | ■■■■■■■■■■■■□■■■■■■■■■■□□■■■■□□□□■□■■■■■■■■■ | EAI6-BGD1 | IO | L1 | 2 (0.8) |
| 35 | 777737777420771 | ■■■■■■■■■■■■□■■■■■■■■■■■■■■■■■□□□■□□□□■■■■■■■ | H3-Ural-1 | EA | L4 | 2 (0.8) |
| 37 | 777737777760771 | ■■■■■■■■■■■■□■■■■■■■■■■■■■■■■■■■■■□□□□■■■■■■■ | T3 | EA | L4 | 11 (4.3) |
| 1688 | 777777403760771 | ■■■■■■■■■■■■■■■■■■■□□□□□□■■■■■■■□□□□■■■■■■■ | LAM | EA | L4 | 2 (0.8) |
| 41 | 777777404760771 | ■■■■■■■■■■■■■■■■■■■□□□□□■□□■■■■■□□□□■■■■■■■ | Turkey | EA | L4 | 5 (1.9) |
| 121 | 777777775720771 | ■■■■■■■■■■■■■■■■■■■■■■■■■□■■■■□■□□□□■■■■■■■ | H3 | EA | L4 | 4 (1.6) |
| 817 | 777777777420731 | ■■■■■■■■■■■■■■■■■■■■■■■■■■■■□□□■□□□□■■■□■■■ | H3-Ural-1 | EA | L4 | 2 (0.8) |
| 777 | 777777777420771 | ■■■■■■■■■■■■■■■■■■■■■■■■■■■■□□□■□□□□■■■■■■■ | H3-Ural-1 | EA | L4 | 2 (0.8) |
| 134 | 777777777720631 | ■■■■■■■■■■■■■■■■■■■■■■■■■■■■■■□■□□□□■■□□■■■ | H3 | EA | L4 | 2 (0.8) |
| 52 | 777777777760731 | ■■■■■■■■■■■■■■■■■■■■■■■■■■■■■■■■□□□□■■■□■■■ | T2 | EA | L4 | 5 (1.9) |
| 53 | 777777777760771 | ■■■■■■■■■■■■■■■■■■■■■■■■■■■■■■■■□□□□■■■■■■■ | T | EA | L4 | 32 (12.4) |
| 54 | 777777777763771 | ■■■■■■■■■■■■■■■■■■■■■■■■■■■■■■■■□□■■■■■■■■■ | Manu2 | EA | L4 | 9 (3.5) |

KBBN: knowledge based Bayesian network; CBN: conformal Bayesian network; SIT: shared international type; EA: Euro-American; EAI: East-African-Indian; IO: Indio-Oceanic. * Supported by SNP typing (Firdessa et al 2013)

**Table 3. Spoligotype descriptions of all orphan or new patterns (n = 26) that were identified among clinical samples collected from both PTB and TBLN patients recruited at selected health facilities in Ethiopia in the year 2016/17.**

| No | Spoligotype pattern of shared SIT strains | | Lineage classification based on | | | # of isolates |
|---|---|---|---|---|---|---|
| | Octal code | Binary format (presence (black) or absence (white) of 43 spacers) | KBBN | CBN | SNP-based prediction* | |
| 1 | 000001777020771 | □□□□□□□□□□□□□□□□□■■■■■■■■■■□□□□■□□□□■■■■■■■ | T1-RUS2 | EA | L4 | 1 |
| 2 | 037677560020771 | □□□□■■■■■■■□■■■■■■■□■■■□□□□□□□□■□□□□■■■■■■■ | H1 | EA | L4 | 1 |
| 3 | 101774000000000 | □□■□□□□□■■■■■■■■□□□□□□□□□□□□□□□□□□□□□□□□□□□ | ZERO | EA | L4 | 1 |
| 4 | 403000377760771 | ■□□□□□□■■□□□□□□□□□□■■■■■■■■■■■■■□□□□■■■■■■■ | T1-RUS2 | EA | L4 | 1 |
| 5 | 477777757000771 | ■□□■■■■■■■■■■■■■■■■■■■□■■■■□□□□□□□□□■■■■■■■ | H4-Ural-2 | EA | L4 | 1 |
| 6 | 503777740003171 | ■□■□□□□■■■■■■■■■■■■■■■□□□□□□□□□□□□■■□□■■■■■ | CAS1-Delhi | EAI | L3 | 1 |
| 7 | 511777400003171 | ■□■□□■□□■■■■■■■■■■■□□□□□□□□□□□□□□□■■□□■■■■■ | CAS | EAI | L3 | 1 |
| 8 | 555777437740171 | ■□■■□■■□■■■■■■■■■■■□□□■■■■■■■■■□□□□□□□■■■■■ | T | EA | L4 | 1 |
| 9 | 603777700003771 | ■■□□□□□■■■■■■■■■■■■■■□□□□□□□□□□□□□■■■■■■■■■ | CAS1-Delhi | EAI | L3 | 1 |
| 10 | 676777660760771 | ■■□■■■■■□■■■■■■■■■■■□■■□□□□■■■■■□□□□■■■■■■■ | T | EA | L4 | 1 |
| 11 | 703737740003571 | ■■■□□□□■■■■■□■■■■■■■■■□□□□□□□□□□□□■■■□■■■■■ | CAS1-Delhi | EAI | L3 | 1 |
| 12 | 703777700001171 | ■■■□□□□■■■■■■■■■■■■■■□□□□□□□□□□□□□□■□□■■■■■ | CAS1-Delhi | EAI | L3 | 2 |
| 13 | 703777740001171 | ■■■□□□□■■□□■■■■■■■■■■■□□□□□□□□□□□□■■□□■■■■■ | CAS1-Delhi | EAI | L3 | 1 |
| 14 | 703777740003171 | ■■■□□□□■■■■■□■■■■■■■■■■□□□□□□□□□□□■■■■■■■■■ | CAS1-Delhi | EAI | L3 | 1 |
| 15 | 703777740003771 | ■■■□□□□■■■■■■■■■□□□□□□■□□□□□□□□□□□□□□□□□■■■ | CAS1-Delhi | EAI | L3 | 1 |
| 16 | 703777747776771 | ■■■□□□□■■■■■■■■■■■■■■■□□■■■■■■■■■■■□■■■■■■■ | Manu1 | EA | L4 | 1 |
| 17 | 711777740003171 | ■■■□□□□□■■■■■■■■■■■■■■□□□□□□□□□□□□■■□□■■■■■ | CAS1-Delhi | EAI | L3 | 1 |
| 18 | 773777776000771 | ■■■■■■□■■■■■■■■■■■■■■■■■■□□□□□□□□□□■■■■■■■ | H3-Ural-1 | EA | L4 | 1 |
| 19 | 776737737760771 | ■■■■■■■■□■■■□■■■■■■■■□■■■■■■■■■■□□□□■■■■■■■ | T3 | EA | L4 | 1 |
| 20 | 777000277760771 | ■■■■■■■■■□□□□□□□□□□■□■■■■■■■■■■■□□□□■■■■■■■ | T3-ETH | EA | L4 | 1 |
| 21 | 777001777760771 | ■■■■■■■■■□□□□□□□□■■■■■■■■■■■■■■■□□□□■■■■■■■ | T3-ETH | EA | L4 | 1 |
| 22 | 777737401760771 | ■■■■■■■■■■■■□■■■■■■□□□□□□□■■■■■■□□□□■■■■■■■ | LAM5 | EA | L4 | 1 |
| 23 | 777737777760000 | ■■■■■■■■■■■■□■■■■■■■■■■■■■■■■■■■□□□□□□□□□□□ | X2 | EA | L4 | 1 |
| 24 | 777777401760771 | ■■■■■■■■■■■■■■■■■■■□□□□□□□■■■■■■□□□□■■■■■■■ | LAM | EA | L4 | 1 |
| 25 | 777777777420571 | ■■■■■■■■■■■■■■■■■■■■■■■■■■■■□□□■□□□□■□■■■■■ | H3-Ural-1 | EA | L4 | 1 |
| 26 | 777777777600631 | ■■■■■■■■■■■■■■■■■■■■■■■■■■■■■□□□□□□□■■□□■■■ | H3 | EA | L4 | 1 |

KBBN: knowledge based Bayesian network; CBN: conformal Bayesian network; SIT: shared international type; EA: Euro-American; EAI: East-African-Indian; IO: Indio-Oceanic. * Supported by SNP typing (Firdessa et al 2013

According to the CBN analysis, 97.3% of the total isolates belonged to two major lineages, EA (61.6%) and EAI (35.7%). On the basis of SNP-based genome-wide phylogeny analysis, these lineages are commonly known as L4 and L3, respectively [23]. The remaining 7/258 (2.7%) were represented by IO (L1) and AFRI (L7), each with three strains, and one with the typical Beijing (L2) spoligotype pattern (Fig 1).

**Fig 1. Proportion of major *Mycobacterium tuberculosis* lineages circulating within peri-urban and urban areas in Ethiopia.** Others' include L7 (AFRI), L2 (Beijing), and L1 (IO)

The alternative KBBN classification showed a predominance of the CAS (34.9%) sub-lineage among strains defined as L3. T (15.9%), T3-ETH (15.1%) and Haarlem (10.9%) were the most common sub-lineages of L4. There was a significant difference in geographical distribution between strain types; all LAM families of L4 (LAM, LAM3 and LAM5) were observed in the northern part of the country (Gondar and Mekele). Similarly, the CAS families (L3), which were highly dominant in the Gondar area, were rather rare around Hawassa. The Manu, Haarlem and T families (all of L4) accounted for the majority of strains identified in the Hawassa region (Fig 2).

**Fig 2. KBBN based classification *of Mycobacterium tuberculosis* sub-lineages circulating within peri-urban and urban areas in Ethiopia.**

**Note:** H1, H3, H3-Ural-1 and H4-Ural-2 were classified as **'**Haarlem'; **'**LAM' include LAM3 and LAM5; Manu represented for Manu1 and Manu2. **'**Others' include the following types: T2, Turkey, T1-RUS2, AFRI (Ethiopian), Beijing, EAI4-VNM and EAI6-BGD1

## Factors associated with strain clustering and predominance

A multivariable analysis (Table 4) showed that as compared to Gondar, rate of clustering in Mekele and Hawassa was more than two and three fold higher, with adjusted OR and 95% CI of 2.71 (1.16, 6.34), and 3.56 (1.09,11.63), respectively. After controlling for the effect of regional variations, adjusted estimates generated from the final model (hierarchical logistic regression analysis) showed that the rate of strain clustering was inversely associated with TB-HIV co-infection and comorbidity with other chronic illnesses. As shown in Table 4, TB-HIV co-infected [0.16 (0.05, 0.47)] individuals and those who had any other concomitant chronic disease [0.16 (0.05, 0.47)] were less likely to have clustered strains as compared to patients diagnosed for only TB disease. Table 4

**Table 4. Conventional and Hierarchical (Multi-level) logistic regression modeling methods were used to identify factors associated with strain clustering.**

| Factor variables | Proportion of cases n (%) | | Estimates of three different logistic regression analysis | | |
|---|---|---|---|---|---|
| | | | Bivariable | Multivariable | Hierarchical |
| | Clustered | Unique | COR (95% CI) | AOR (95% CI) | AOR (95% CI) |
| **Region** | | | | | |
| Gondar | 77(38.3) | 34(59.6) | Ref | Ref | Level-I factor |
| Hawassa | 37(18.4) | 5 (8.8) | 3.17 (1.14,8.79)* | 3.56 (1.09,11.63)* | |
| Mekele | 51(25.4) | 10(17.5) | 2.19 (0.99,4.82) | 2.71 (1.16,6.34)* | |
| Addis Ababa | 36(17.9) | 8(14.0) | 1.93 (0.81,4.59) | 2.42 (0.84,7.01) | |
| **Diagnosis** | | | | | |
| PTB | 127(63.2) | 36(63.2) | Ref | Ref | Ref |
| TBLN | 74(36.8) | 21(36.8) | 0.97 (0.53,1.79) | 0.52 (0.24,1.15) | 0.58 (0.27,1.23) |

| | | | | | |
|---|---|---|---|---|---|
| **HIV co-infection** | | | | | |
| No | 191(95.0) | 47(82.5) | Ref | Ref | Ref |
| Yes | 10 (5.0) | 10(17.5) | **0.27 (0.11,0.71)**** | **0.16 (0.05,0.50)**** | **0.16 (0.05, 0.47)***** |
| **Co-morbidity of Chronic illness** | | | | | |
| No | 134(66.7) | 28(49.1) | Ref | Ref | Ref |
| Yes | 67(33.3) | 29(50.9) | **0.50 (0.27,0.91)*** | 0.50 (0.25,1.01) | **0.46 (0.23,0.91)*** |
| **Hemoptysis** | | | | | |
| No | 167(83.1) | 42 (75.0) | Ref | Ref | Ref |
| Yes | 34(16.9) | 14 (25.0) | 0.61 (0.30,1.24) | 0.50 (0.22,1.16) | 0.55 (0.24, 1.25) |
| **TB lineage** | | | | | |
| L3 (EAI) | 76(37.8) | 16(28.1) | Ref | Ref | Ref |
| L4 (EA) | 121(60.2) | 38(66.7) | 0.69 (0.36,1.32) | **0.42 (0.20,0.90)*** | 0.49 (0.23, 1.04) |
| Others | 4 (2.0) | 3 (5.3) | 0.28 (0.06,1.38) | 0.25 (0.04,1.48) | 0.25 (0.04, 1.44) |

The cut-off point for statistical significance ($\alpha$) is represented by: < 0.05 = *; < 0.01 = **; < 0.001 = ***

Note: Three different statistical analyses method was performed and presented here for comparison, but the key findings are reported based on adjusted estimates generated from the final multilevel statistical analysis method (Hierarchical modeling)

A second multivariable analysis was performed in relation to the clinical characteristics of the two most predominant lineages (L3 and L4). As shown in Table 5, in comparison to L4 strains of *M. tuberculosis*, the odds for TBLN cases infected with L3 was three and half fold [3.47 (1.45, 8.29)] higher than PTB patients. Active TB disease due to L3 strains was significantly associated with HIV-TB co-infection [2.84 (1.61, 5.55)], but less likely to be associated with concomitant chronic disease [0.46 (0.25, 0.87)], as compared to L4. Table 5

**Table 5. Logistic regression analysis used to measure and compare the clinical characteristics associated with active TB disease caused by L3 versus L4, the two most dominant *Mycobacterium tuberculosis* lineages identified in the study.**

| **Clinical characteristics** | **Proportion of Cases: n (%)** | | **Bivariable analysis** | | **Multivariable analysis** | |
|---|---|---|---|---|---|---|
| | Lineage 3 | Lineage 4 | COR (95% CI) | P-value | AOR (95% CI) | P-value |
| **Region** | | | | | | |
| Addis Ababa | 12(13.0) | 32(20.1) | Ref | | Ref | |
| Gondar | 54(58.7) | 53(33.3) | 2.77 (1.29,5.95) | 0.009 | 5.24 (2.03,13.51) | < 0.001 |
| Hawassa | 1 (1.1) | 41(25.8) | 0.07 (0.01,0.53) | 0.010 | 0.11 (0.01,0.95) | 0.044 |
| Mekele | 25(27.2) | 33(20.8) | 2.02 (0.87,4.69) | 0.102 | 4.28 (1.52,11.99) | 0.006 |
| **Gender** | | | | | | |
| Male | 56(60.9) | 88(55.3) | Ref | | Ref | |
| Female | 36(39.1) | 71(44.7) | 0.79 (0.47,1.33) | 0.371 | 0.91 (0.48,1.72) | 0.781 |
| **Diagnosis** | | | | | | |
| PTB | 53(57.6) | 107(67.3) | Ref | | Ref | |
| TBLN | 39(42.4) | 52(32.7) | 1.5 (0.88,2.55) | 0.134 | 3.47 (1.45,8.29) | 0.005 |
| **HIV co-infection** | | | | | | |
| No | 81(88.0) | 151(95.0) | Ref | | Ref | |

| | | | | | | |
|---|---|---|---|---|---|---|
| Yes | 11(12.0) | 8 (5.0) | 2.93 (1.09,7.85) | 0.033 | 2.84 (1.61,5.55) | 0.027 |
| **Comorbidity of Chronic illness** | | | | | | |
| No | 62(67.4) | 95(59.7) | Ref | | Ref | |
| Yes | 30(32.6) | 64(40.3) | 0.73 (0.43,1.25) | 0.252 | 0.46 (0.25,0.87) | 0.016 |
| **Taking prescribed Medication** | | | | | | |
| No | 55(59.8) | 117(73.6) | Ref | | Ref | |
| Yes | 37(40.2) | 42(26.4) | 1.86 (1.08,3.21) | 0.026 | 1.67 (0.83,3.36) | 0.152 |
| **Persistent Cough** | | | | | | |
| No | 19(20.7) | 32(20.1) | Ref | | Ref | |
| Yes | 73(79.3) | 127(79.9) | 0.94 (0.49,1.78) | 0.844 | 1.03 (0.41,2.61) | 0.944 |
| **Hemoptysis** | | | | | | |
| No | 74(80.4) | 129(81.6) | Ref | | Ref | |
| Yes | 18(19.6) | 29(18.4) | 1.08 (0.56,2.08) | 0.813 | 2.10 (0.90,4.87) | 0.085 |
| **Weight loss** | | | | | | |
| No | 12(13.0) | 27(17.0) | Ref | | Ref | |
| Yes | 80(87.0) | 132(83.0) | 1.37 (0.66,2.86) | 0.397 | 1.00 (0.41,2.47) | 0.997 |

# Discussion

Despite previous achievements in reducing national TB morbidity and mortality [24], summarized reports of data from the global burden of TB diseases in the last two decades have shown a declined rate in reducing the prevalence and mortality ratio in Ethiopia. Essentially, there has been a higher rate of new TB cases (incidence) in the last few years than what was expected from the previous trend [25, 26]. Understandably, at national level, some population groups have likely contributed more to such a TB incidence rate than other groups. Particularly, the risk of TB transmission around urban areas is known to be higher than among sparsely populated societies and rural communities [12, 17]. Because of the simultaneously ongoing expansion of urbanization and emerging socio-economic conditions around urban areas in Ethiopia (increasing population size and density e.g. through expanding slums, congregation into condominiums, growing manufacturing and service sector), the pattern of TB transmission among those living and working in the urban and peri-urban areas is postulated to differ in strain diversity and clustering from that in the general population [17], the majority (85%) of which are rural communities.

Accordingly, a diverse range of strains of *M. tuberculosis* lineages, many previously not registered in spoligotyping databases, continue to circulate and maintain a high rate of transmission of TB in Ethiopia. Similarly, as would be expected, the observed diversified type of *M. tuberculosis* strain and lineage distribution in the current study closely matched with studies analyzed in the two most recent TB reviews that showed specific lineage predominance across different geographical locations in Ethiopia [17, 27]. This means, the same two major lineages, L4 and L3 (Fig 1), were predominant [17, 18, 27], as were the five most common SIT patterns (Table 2) [17, 28-31]. As shown in Figs 1 and 2, the observed significant difference in proportions of strain types across the four study sites, has also been noted from previous studies in Ethiopia [17, 27]. Those less prevalent *M. tuberculosis* lineages, which included the Ethiopian (L7), the Beijing (L2), and the IO (L1) lineages, were identified from samples collected at sites located in the northern regions (Gondar and Mekele). Strains of L7,

which was first reported by Firdessa et al [16, 28, 29, 32] and that seem highly confined to Ethiopia, remain more prevalent in the north of the country. The two SIT patterns (SIT1729 and SIT910) that we identified in this region are the same as for those strains that were previously classified as L7 [21, 28].

Beside difference in geographical locations, risk of transmission and TB disease progression is likely to depend on the interactions of various factors related to strain type and host immunity [21]. Genetic difference has been shown to have an impact on the extent of TB transmission; thus strains from TB lineages referred to as 'modern' lineages (L2-L4) are more transmissible than other MTBC strains [33]. While comparing the two most predominant *M. tuberculosis* lineages, a multivariate logistic regression analysis showed a relatively lower rate of clustering among L4 as compared to L3 strains. Altogether, a predominance of known SIT patterns resulted in a considerably higher rate of overall strain clustering (0.78) in our study, with slight difference across the study regions (Table 4). That was significantly higher as compared to earlier Ethiopian studies (2005–2018) reviewed by Mekonnen et al. (2019) with pooled clustering rate and 95% CI of 0.41(0.32 – 0.50) [27]. Despite limited discriminatory power of the spoligotyping method, an increased rate of *M. tuberculosis* transmission is generally inferred by comparing clustered genotyping patterns of clinical isolates from a given epidemiological setting [6]. By contrast, cases with isolates of a unique pattern could be considered to have resulted from reactivation of latent infection or were else presumably acquired outside of the study population [22]. Apparently, higher risk of primary exposure or an increased rate of TB transmission in endemic settings has often been associated with the presence of more infectious PTB cases [1]. On the other hand, poor host immunity has been linked with endogenous reactivation of latent infection and could have greater contribution to the development of TBLN or disseminated TB [31]. However, as previously reported by others in several studies, we also did not observe any difference in clustering rate with respect to site of infection [1, 27-29]. This might be because of limited power of the study that could not control for all possible effects of confounding factors. It is interesting to note that after adjusting for the effect of regional variations, the likelihood of clustering was significantly lower among HIV co-infected patients and those who had any other concomitant chronic diseases.

Although, the differences in strain virulence and immunogenicity have been investigated in experimental studies, whether this phenotypic variation plays a role in human disease remains unclear [34]. Therefore, it is believed that investigating the clinical epidemiology of dominant *M. tuberculosis* lineages among host populations would allow understanding of possible host-pathogen interaction. In this regard, one of the findings that emerged from this study is that clinical factors, which are often associated with host immunity, differed significantly between L3 and L4, the two most dominant lineages. According to the multivariate analysis (Table 5), the likelihood of detecting L3 among TBLN cases and HIV co-infected patients was significantly higher than for L4. Whereas, summary report that describes the global database has shown a higher rate of CAS (L3) infection among HIV cases than other widely prevalent sub-lineages [21]. The observed discrepancy might be due to the interaction effect of sub-lineages that were grouped in one category or the possibility of co-infection within the same host. Although it is often associated with host immunity, Osório et al. (2018) stated that due to selective advantage of extrinsic factors, within-host bacterial diversity seems to contribute to difference in disease progression [5]. For example, certain groups of L4 strains are found to be more virulent in terms of disease severity and to display higher rates of human-to-human transmission, but only at some specific geographical locations [33]. In favor of that, and as compared to L4, the current study identified significantly lower rate of L3 strains among TB cases diagnosed with other concomitant chronic illnesses (Table 5). Certainly, any immune-compromised condition and HIV interferes with bacterial virulence and might lead to endogenous reactivation [1, 13], suggesting that less virulent MTBC species could progress to active TB disease in immune-compromised patients. For example, TB patients infected with *M. africanum* were more likely to be older, HIV

infected, and severely malnourished than those infected with *M. tuberculosis* [35]. Although the mechanisms are not yet clear, the influence of bacterial and host genotype on the development of different forms of TB in humans is well documented [30]. In this regard, the findings observed in this study seem to agree with others that suggested a possible relationship between L3 and EPTB disease [31, 36]. Correspondingly, a significantly higher rate of PTB was often associated with L4, while more EPTB disease, such as TB meningitis and TBLN, was attributed to L3 [37-39].

Generally, because of a complex network related with many other proximal and distal determinants, *M. tuberculosis* strain clustering or lineage specific effects on disease presentations may not always be fully explained by some particular risk factors and it is difficult to quantify the biological effect using numerical estimates [40]. Henceforth, most of the previously reported epidemiological studies in humans have come up with inconsistent findings [33]. It is known that heterogeneity is a defining feature of TB, which is certainly common in molecular studies [40]. However, although the need for additional clinical evidence is obvious, one can state that disease phenotypes can possibly be determined by genotype features of specific strains, suggesting that different *M. tuberculosis* lineages could be more frequently present in specific clinical phenotypes and disease presentations than in others [33].

# Limitation

Spoligotyping has its limitations and may not truly detect the ongoing change (genetic difference) for investigating transmission networks [7]. Alternative molecular diagnostic tools such as MIRU-VNTR and especially whole genome sequencing have been shown to have better discriminatory power for investigating strain clustering and to confirm the ongoing rate of active TB disease transmission [7, 28, 41]. Similarly, the fairly small sample size, uneven representation of strains from the study sites, and further categorization into different levels of factor variables, has reduced the power of our statistical analysis. Hence, the numerical estimates may not truly imitate the biological interaction or effect modification on host related factors and specific *M. tuberculosis* lineages. However, the strength of the study is in that it recognizes not only systematic and measurement errors, but also selection and recall bias and cautions in the interpretation of findings. It has minimized anticipated measurement errors and known confounding effects. For instance, alongside with internal quality control procedures for the identification of lineages, SITVIT patterns were compared with alternative lineage classifications generated from linked databases (KBBN and CBN) and further verified using SNP based predications. In addition, the multivariate analysis has considered the expected effect of regional variation on TB lineage predominance and this was therefore further triangulated with clinical and demographic data and adjusted for known host related factors.

# Conclusion and Recommendation

A diverse *M. tuberculosis* population structure, dominated by several endemic lineages and sub-lineages, were found to be circulating in four peri-urban regions of Ethiopia. Despite differences in geographical variations, we investigated the contribution of bacterial genotype and host related factors associated with rate of strain clustering and predominance. Rate of strain clustering was relatively higher among patients infected with L3 strain of *M. tuberculosis* as compared to L4; and it was inversely associated with TB-HIV co-infection and comorbidity with other chronic illnesses. A second multivariable analysis indicated that active TB disease due L3 strains were three times higher among TBLN patients and it was more likely to be associated with TB-HIV co-

infection; but inversely associated with other concomitant chronic disease, as compared to L4. Although there is some other supportive evidence, it is difficult to be conclusive on a fixed categorical relationship between strain sub-lineages and disease type in this study because of the complex pathogenesis of TB and the predisposing environmental factors not considered here, that all together contribute to the continuous flux in the spectrum of TB infection and disease progression. However, the current findings add up to previous indications of a probable relationship worth investigating further. Indeed, further large scale studies using advanced molecular techniques like whole genome sequencing could reveal the degree to which this genetic variation influences disease epidemiology and phenotype in different population groups over time as the dynamics of socioeconomic transformations exert pressure on how people live and interact.

# Acknowledgments

We would like to forward our appreciation to supportive staff at the Armauer Hansen Research Institute and all members of the ETHICOBOTS project who had a great contribution to the success of this study. Besides, we would like to extend our acknowledgment to the University of Gondar and the academic staff of the public health institute.

The members of the Ethiopia Control of Bovine Tuberculosis Strategies (ETHICOBOTS) consortium are: Abraham Aseffa, Adane Mihret, Bamlak Tessema, Bizuneh Belachew, Eshcolewyene Fekadu, Fantanesh Melese, Gizachew Gemechu, Hawult Taye, Rea Tschopp, Shewit Haile, Sosina Ayalew, Tsegaye Hailu, all from Armauer Hansen Research Institute, Ethiopia; Rea Tschopp from Swiss Tropical and Public Health Institute, Switzerland; Adam Bekele, Chilot Yirga, Mulualem Ambaw, Tadele Mamo, Tesfaye Solomon, all from Ethiopian Institute of Agricultural Research, Ethiopia; Tilaye Teklewold from Amhara Regional Agricultural Research Institute, Ethiopia; Solomon Gebre, Getachew Gari, Mesfin Sahle, Abde Aliy, Abebe Olani, Asegedech Sirak, Gizat Almaw, Getnet Mekonnen, Mekdes Tamiru, Sintayehu Guta, all from National Animal Health Diagnostic and Investigation Centre, Ethiopia; James Wood, Andrew Conlan, Alan Clarke, all from Cambridge University, United Kingdom; Henrietta L. Moore and Catherine Hodge, both from University College London, United Kingdom; Constance Smith at University of Manchester, United Kingdom; R. Glyn Hewinson, Stefan Berg, Martin Vordermeier, Javier Nunez-Garcia, all from Animal and Plant Health Agency, United Kingdom; Gobena Ameni, Berecha Bayissa, Aboma Zewude, Adane Worku, Lemma Terfassa, Mahlet Chanyalew, Temesgen Mohammed, Yemisrach Zeleke, all from Addis ababa University, Ethiopia.

# References

1. Srilohasin P, Chaiprasert A, Tokunaga K, Nishida N, Prammananan T, Smittipat N, et al. Genetic diversity and dynamic distribution of Mycobacterium tuberculosis isolates causing pulmonary and extrapulmonary tuberculosis in Thailand. J Clin Microbiol. 2014;52(12):4267-74.

2. Gonzalo-Asensio J, Malaga W, Pawlik A, Astarie-Dequeker C, Passemar C, Moreau F, et al. Evolutionary history of tuberculosis shaped by conserved mutations in the PhoPR virulence regulator. Proceedings of the National Academy of Sciences. 2014;111(31):11491-6.

3. Gagneux S. Host-pathogen coevolution in human tuberculosis. Philosophical Transactions of the Royal Society B: Biological Sciences. 2012;367(1590):850-9.

4. Reynaud Y, Millet J, Rastogi N. Genetic Structuration, Demography and Evolutionary History of Mycobacterium tuberculosis LAM9 Sublineage in the Americas as Two Distinct Subpopulations Revealed by Bayesian Analyses. PLoS One. 2015;10(10):e0140911.

5. Bastos HN, Osório NS, Gagneux S, Comas I, Saraiva M. The Troika host–pathogen–extrinsic factors in tuberculosis: modulating inflammation and clinical outcomes. J Frontiers in immunology. 2018;8:1948.

6. Reiling N, Homolka S, Walter K, Brandenburg J, Niwinski L, Ernst M, et al. Clade-specific virulence patterns of Mycobacterium tuberculosis complex strains in human primary macrophages and aerogenically infected mice. mBio. 2013;4(4).

7. Meehan CJ, Moris P, Kohl TA, Pečerska J, Akter S, Merker M, et al. The relationship between transmission time and clustering methods in Mycobacterium tuberculosis epidemiology. J EBioMedicine. 2018;37:410-6.

8. Van der Spuy G, Warren R, Richardson M, Beyers N, Behr M, Van Helden P. Use of genetic distance as a measure of ongoing transmission of Mycobacterium tuberculosis. Journal of Clinical Microbiology. 2003;41(12):5640-4.

9. Borgdorff M, Van Soolingen D. The re-emergence of tuberculosis: what have we learnt from molecular epidemiology? J Clinical Microbiology Infection. 2013;19(10):889-901.

10. Pareek M, Evans J, Innes J, Smith G, Hingley-Wilson S, Lougheed KE, et al. Ethnicity and mycobacterial lineage as determinants of tuberculosis disease phenotype. J Thorax. 2013;68(3):221-9.

11. Ferraris DM, Miggiano R, Rossi F, Rizzi M. Mycobacterium tuberculosis Molecular Determinants of Infection, Survival Strategies, and Vulnerable Targets. Pathogens. 2018;7(1).

12. Mekonnen A, Merker M, Collins JM, Addise D, Aseffa A, Petros B, et al. Molecular epidemiology and drug resistance patterns of Mycobacterium tuberculosis complex isolates from university students and the local community in Eastern Ethiopia. PLoS One. 2018;13(9):e0198054.

13. Suzana S, Shanmugam S, Uma Devi KR, Swarna Latha PN, Michael JS. Spoligotyping of Mycobacterium tuberculosis isolates at a tertiary care hospital in India. Tropical medicine & international health : TM & IH. 2017;22(6):703-7.

14. Bedewi Z, Worku A, Mekonnen Y, Yimer G, Medhin G, Mamo G, et al. Molecular typing of Mycobacterium tuberculosis complex isolated from pulmonary tuberculosis patients in central Ethiopia. BMC Infect Dis. 2017;17(1):184.

15. Berg S, Schelling E, Hailu E, Firdessa R, Gumi B, Erenso G, et al. Investigation of the high rates of extrapulmonary tuberculosis in Ethiopia reveals no single driving factor and minimal evidence for zoonotic transmission of Mycobacterium bovis infection. BMC Infect Dis. 2015;15:112.

16. Yimer SA, Norheim G, Namouchi A, Zegeye ED, Kinander W, Tonjum T, et al. Mycobacterium tuberculosis lineage 7 strains are associated with prolonged patient delay in seeking treatment for pulmonary tuberculosis in Amhara Region, Ethiopia. J Clin Microbiol. 2015;53(4):1301-9.

17. Tulu B, Ameni G. Spoligotyping based genetic diversity of Mycobacterium tuberculosis in Ethiopia: a systematic review. J BMC infectious diseases. 2018;18(1):140.

18. Tilahun M, Ameni G, Desta K, Zewude A, Yamuah L, Abebe M, et al. Molecular epidemiology and drug sensitivity pattern of Mycobacterium tuberculosis strains isolated from pulmonary tuberculosis patients in and around Ambo Town, Central Ethiopia. PLoS One. 2018;13(2):e0193083.

19. Berg S, Firdessa R, Habtamu M, Gadisa E, Mengistu A, Yamuah L, et al. The burden of mycobacterial disease in ethiopian cattle: implications for public health. PLoS One. 2009;4(4):e5068.

20. Kamerbeek J, Schouls L, Kolk A, van Agterveld M, van Soolingen D, Kuijper S, et al. Simultaneous detection and strain differentiation of Mycobacterium tuberculosis for diagnosis and epidemiology. J Clin Microbiol. 1997;35(4):907-14.

21. Couvin D, David A, Zozio T, Rastogi N, Evolution. Macro-geographical specificities of the prevailing tuberculosis epidemic as seen through SITVIT2, an updated version of the Mycobacterium tuberculosis genotyping database. J Infection, Genetics. 2019;72:31-43.

22. Kato-Maeda M, Metcalfe JZ, Flores L. Genotyping of Mycobacterium tuberculosis: application in epidemiologic studies. Future Microbiol. 2011;6(2):203-16.

23. Comas I, Homolka S, Niemann S, Gagneux S. Genotyping of genetically monomorphic bacteria: DNA sequencing in Mycobacterium tuberculosis highlights the limitations of current methodologies. PLoS One. 2009;4(11):e7815.

24. Glaziou P, Floyd K, Raviglione MC, editors. Global epidemiology of tuberculosis. Seminars in respiratory and critical care medicine; 2018: Thieme Medical Publishers.

25. Deribew A, Deribe K, Dejene T, Tessema GA, Melaku YA, Lakew Y, et al. Tuberculosis Burden in Ethiopia from 1990 to 2016: Evidence from the Global Burden of Diseases 2016 Study. Ethiop J Health Sci. 2018;28(5):519-28.

26. Kyu HH, Maddison ER, Henry NJ, Ledesma JR, Wiens KE, Reiner Jr R, et al. Global, regional, and national burden of tuberculosis, 1990–2016: results from the Global Burden of Diseases, Injuries, and Risk Factors 2016 Study. J The Lancet Infectious Diseases. 2018;18(12):1329-49.

27. Mekonnen D, Derbie A, Chanie A, Shumet A, Biadglegne F, Kassahun Y, et al. Molecular epidemiology of M. tuberculosis in Ethiopia: A systematic review and meta-analysis. Tuberculosis (Edinburgh, Scotland). 2019;118:101858.

28. Firdessa R, Berg S, Hailu E, Schelling E, Gumi B, Erenso G, et al. Mycobacterial lineages causing pulmonary and extrapulmonary tuberculosis, Ethiopia. Emerg Infect Dis. 2013;19(3):460-3.

29. Nuru A, Mamo G, Worku A, Admasu A, Medhin G, Pieper R, et al. Genetic Diversity of Mycobacterium tuberculosis Complex Isolated from Tuberculosis Patients in Bahir Dar City and Its Surroundings, Northwest Ethiopia. BioMed Research International. 2015;2015:174732.

30. Coscolla M, Gagneux S, editors. Consequences of genomic diversity in Mycobacterium tuberculosis. Seminars in immunology; 2014: Elsevier.

31. Khandkar C, Harrington Z, Jelfs PJ, Sintchenko V, Dobler CC. Epidemiology of Peripheral Lymph Node Tuberculosis and Genotyping of M. tuberculosis Strains: A Case-Control Study. PLoS One. 2015;10(7):e0132400.

32. Belay M, Ameni G, Bjune G, Couvin D, Rastogi N, Abebe F. Strain diversity of Mycobacterium tuberculosis isolates from pulmonary tuberculosis patients in Afar pastoral region of Ethiopia. BioMed Research International. 2014;2014:238532.

33. Coscolla M. Biological and Epidemiological Consequences of MTBC Diversity. Advances in experimental medicine and biology. 2017;1019:95-116.

34. Coscolla M, Gagneux S. Does M. tuberculosis genomic diversity explain disease diversity? J Drug Discovery Today: Disease Mechanisms. 2010;7(1):e43-e59.

35. de Jong BC, Antonio M, Gagneux S. Mycobacterium africanum--review of an important cause of human tuberculosis in West Africa. PLoS Neglected Tropical Diseases. 2010;4(9):e744.

36. Drain PK, Bajema KL, Dowdy D, Dheda K, Naidoo K, Schumacher SG, et al. Incipient and Subclinical Tuberculosis: a Clinical Review of Early Stages and Progression of Infection. Clin Microbiol Rev. 2018;31(4).

37. Caws M, Thwaites G, Dunstan S, Hawn TR, Lan NT, Thuong NT, et al. The influence of host and bacterial genotype on the development of disseminated disease with Mycobacterium tuberculosis. PLoS Pathogens. 2008;4(3):e1000034.

38. Kong Y, Cave M, Yang D, Zhang L, Marrs C, Foxman B, et al. Distribution of insertion-and deletion-associated genetic polymorphisms among four Mycobacterium tuberculosis phospholipase C genes and associations with extrathoracic tuberculosis: a population-based study. J Journal of clinical microbiology. 2005;43(12):6048-53.

39. Kong Y, Cave M, Zhang L, Foxman B, Marrs C, Bates J, et al. Association between Mycobacterium tuberculosis Beijing/W lineage strain infection and extrathoracic tuberculosis: insights from epidemiologic and clinical characterization of the three principal genetic groups of M. tuberculosis clinical isolates. J Journal of clinical microbiology. 2007;45(2):409-14.

40. Trauer JM, Dodd PJ, Gomes MGM, Gomez GB, Houben RM, McBryde ES, et al. The importance of heterogeneity to the epidemiology of tuberculosis. J Clinical infectious diseases. 2018;69(1):159-66.

41. Takii T, Seki K, Wakabayashi Y, Morishige Y, Sekizuka T, Yamashita A, et al. Whole-genome sequencing-based epidemiological analysis of anti-tuberculosis drug resistance genes in Japan in 2007: Application of the Genome Research for Asian Tuberculosis (GReAT) database. Scientific Reports. 2019;9(1):12823.

# Supportive information

**S1 Table. Spoligotype descriptions and lineage classifications for of all clinical isolates**

Fig 1

Click here to access/download;Figure;Fig 1.tif

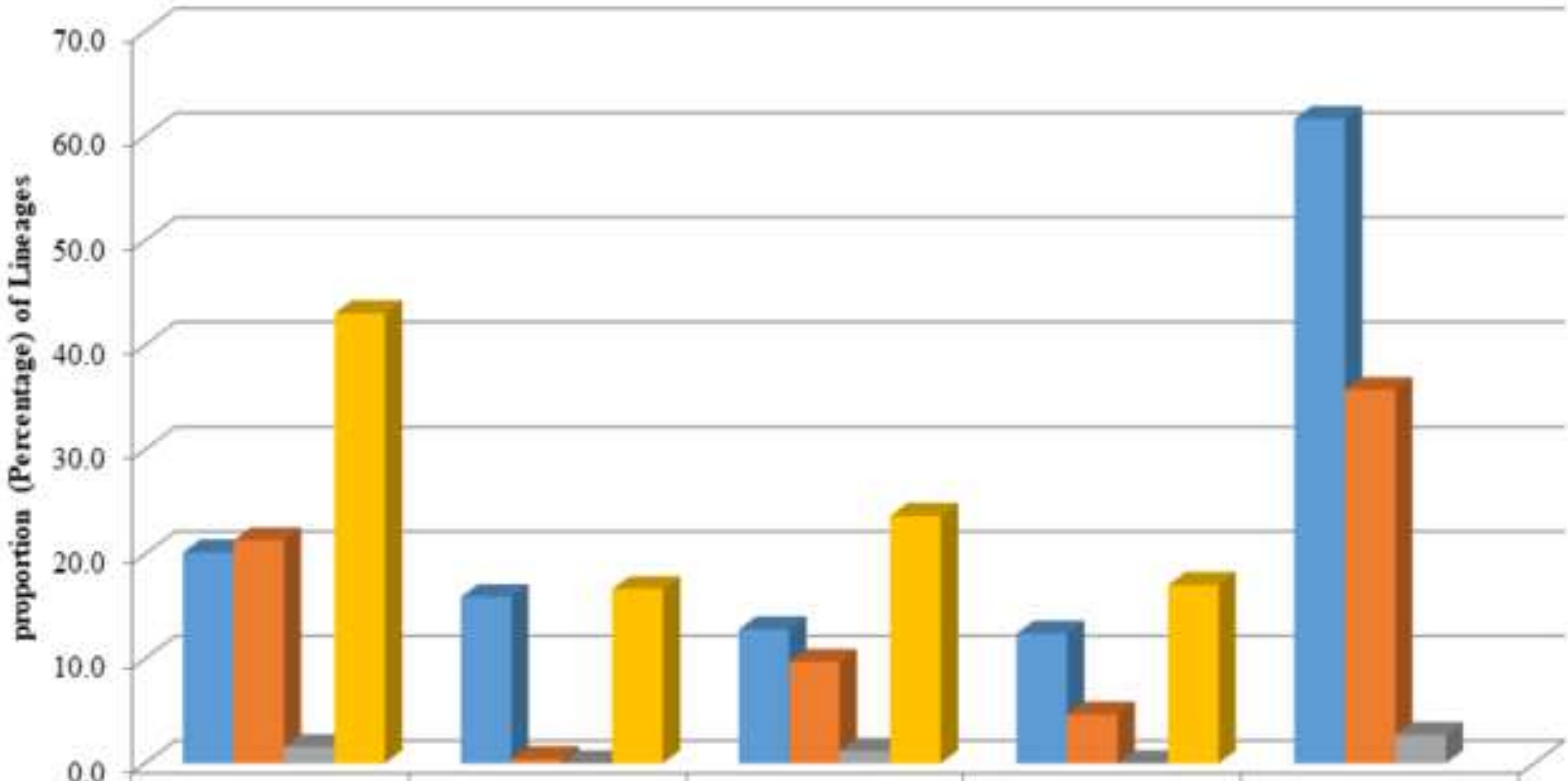


| | Gondar | Hawassa | Mekele | Addis Ababa | Total |
|---|---|---|---|---|---|
| L4 | 20.2 | 15.9 | 12.8 | 12.4 | 61.6 |
| L3 | 21.3 | 0.4 | 9.7 | 4.7 | 35.7 |
| Others | 1.6 | 0.0 | 1.2 | 0.0 | 2.7 |
| Total | 43.0 | 16.7 | 23.6 | 17.1 | |

Fig 2 Click here to access/download;Figure;Fig 2.tif

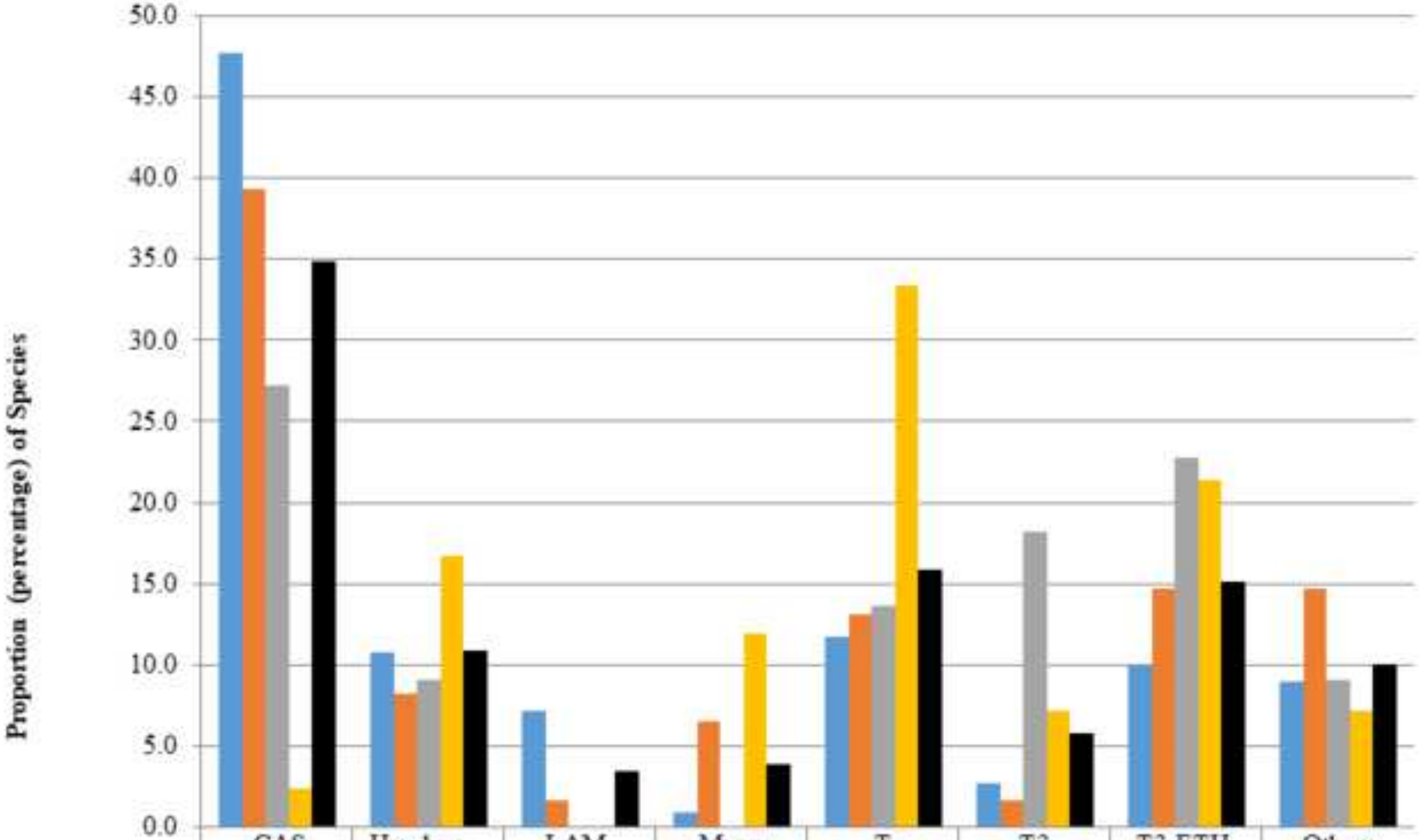


| | CAS | Haarlem | LAM | Manu | T | T3 | T3-ETH | Others |
|---|---|---|---|---|---|---|---|---|
| Gondar | 47.8 | 10.8 | 7.2 | 0.9 | 11.7 | 2.7 | 9.9 | 9.0 |
| Mekele | 39.3 | 8.2 | 1.6 | 6.6 | 13.1 | 1.6 | 14.8 | 14.8 |
| Addis Ababa | 27.3 | 9.1 | 0.0 | 0.0 | 13.6 | 18.2 | 22.7 | 9.1 |
| Hawassa | 2.4 | 16.7 | 0.0 | 11.9 | 33.3 | 7.1 | 21.4 | 7.1 |
| Total | 34.9 | 10.9 | 3.5 | 3.9 | 15.9 | 5.8 | 15.1 | 10.1 |

Click here to access/download
**Supporting Information**
S1 Table.xls
