# Supplementary material for: Epidemiology of Mycobacterium tuberculosis lineages and strain clustering within urban and peri-urban settings in Ethiopia
Source: PLoS One. 2021 Jul 12;16(7):e0253480. doi: 10.1371/journal.pone.0253480 (PMC8274931; doi:10.1371/journal.pone.0253480)
Supplement: Attachment — Submitted filename: Response to Reviewers.docx [file pone.0253480.s006.docx]

**Addis Ababa, May 25, 2021**

I. **Author’s response to comments and points raised by the academic editor.**

The revised version of our manuscript meets PLOS ONE's style requirements, that including:

- File naming; syntax and definition of first coming abbreviation were corrected
- The size of the titled and the abstract reduced as per the required word limit.
- We provide detail ethical statements including approvable number, but genotype analysis and comparison with global databases (SITVIT2) It is free Online database which did not required approval letter.
- We modified Table 1 as per the comments and also formatted other Tables
- In the correct version, we provided PACE corrected figures
- In the analysis section, the role of hierarchical (multi-level) logistic regression model had further explained and all necessary software codes, including major steps for model fitting criteria had provided
- The respective the dataset will be provided while our manuscript is accepted for publication.
- We made the required revision on the conclusion and recommendation section in accordance with comments from the academic editor. Now the clinical and public health implication and the general recommendation has presented in separate paragraphs.
- List of the ETHICOBOTS consortium group authors has been already mentioned in the acknowledgments section of the manuscript and with some correction from previous version.
- There was no any intention to cite a retracted article that was done unknowingly due to technical problems while importing articles from databases. Now excluded those papers and older articles except Kamerbeek, J. et al. (1997) and Berg et al. (2009). This is because we would like to acknowledge the one who develop the original protocol used for the current study.

II. **Comments and respective response to Reviewer #1:**

1. At end of the title there is a punctuation (.) which have to delete

- Corrected

2. In the abstract authors time to time used abbreviation (PTB, TBLN, SITVIT2, SIT) which is difficult to understand by the general reader. Thus, an elaboration should include.

- Corrected and all abbreviation were defined in first place

3. In the Methods of the abstract authors mentioned that they have collected the sample from four different regions in Ethiopia were recruited in the year 2016 and 2017. But why they have publishing this result after 3 years. Need an explanation for that because within this time period lineage of microorganism may change. In addition, it is prescribed to mention the exclusion and inclusion criteria of TB patient’s recruitment.

- The participant recruitment and filed data collection for the particular work-package was completed in the year 2016 and 2017. However, as it is part of the consortium project that integrated with other work-packages, the overall project activities takes more time. Particularly, specimen (Sputum and FNA), preparation and processing that include culturing and molecular typing demand more time. This is mainly because of the fact that most of laboratory inputs (reagents and equipment) were not available in domestic market. Thus, purchasing and material shipments from international market is also required extra time. We believed that once the data collection period has stated, ongoing change in lineage epidemiology may not affect plausibility of the current study.
- Because of the word limit in the abstract section, further descriptions of the study population that include exclusion and inclusion criteria of patient recruitment had mentioned in the methodology section in the main manuscript body.

4. From the title its clear that authors wanted to find out epidemiology and factors associated with strain clustering and lineage predominance but in the introduction they failed to good use of literature review. They should clearly include what are the possible factors that can be associate and with brief introduction of underlying mechanism. For example, they found that L3 M. tuberculosis strains were more likely to be associated with TBLN and TB-HIV co-infection, but what is the possible mechanism of this association and co-infection. Why M. tuberculosis (bacteria) have association with HIV (virus).

- Following the comment of the reviewer, additional literatures were added in the introduction section. Nevertheless, as it is a population based epidemiological study, detailed explanation for biological interaction of specific lineages and disease phenotype is beyond the scope of the study. Indeed, although, it is still difficult to have clear understanding on the possible mechanism of influence of bacterial and host genotype on the development of different forms of TB in humans, we tried to highlight and support with findings reported from basic (biomedical) research.

5. For ethical consideration, need to add reference no. of the ethical clearance

- Corrected! The ethical clearance reference numbers obtained from three different institutes are inserted.

6. In the result section, if (optional) the sequencing data of all strains in different geographic region of Ethiopia is available, a phylogenetic tree can represent the closeness of the strains and also better understanding of lineage.

- Unfortunately, this study was done based on Spoligotyping identification techniques and was limited to perform advanced molecular sequencing technologies- That is clearly mentioned as one of the study limitation.

6. In the discussion, authors need elaborate on what are the underlying factors that contributed to prevail more TB in the urban than rural area.

- In the first paragraph of the discussion section, we mentioned the main underlying factors that contributed to prevail more TB in the urban than rural area

7. In the discussion (Line 275-276), author mentioned unique pattern considered to have resulted from reactivation of latent infection. But what about adaptation of those strains with environment and genetical changes (mutation/polymorphisms)?

- Further explanation has done according to the comment.

III. **Comments and respective response to Reviewer #2:**

It is my pleasure to review such a well written manuscript. The data were rigorously analyzed. It involved a great deal of work and dedication to complete this work and it is clearly visible. However, I would like to ask one question to the authors and that would be on sample size calculation. I could not find any clear description on how the investigators arrived at the sample size used in this analysis. The purposive selection of sites coupled with lack of information on sample size calculation makes it difficult to accept the results of such rigorous analysis and also puts the question of generalizability forward. Are these analyses generalizable to the whole community or the regions? We do not know and this information is vital to the analysis. I am sure the authors will be able to answer the question. If it is an exploratory study and the sample size calculation was not done rigorously. I would request the authors to include this information in the limitations section explicitly to make the readers aware of the fact. I congratulate the authors for their hard work and look forward to see their response. Thanks!

- Thank you for the comments, we totally agreed with all the points raised.

As mentioned in the methodology section, it is a multi-Centre health facility based cross-sectional study where four regional cities including Addis Ababa (the capital city of Ethiopia)	were purposively selected as part of the consortium (ETHICOBOTS) project. The main aim of this project was primarily targeted to estimate prevalence of Zoonotic TB among people working and living around those urban and peri-urban areas. Given that we had calculated a sample size and recruited larger numbers of study participants	from selected health facilities.

However, as there was low rate of culture positive samples, which is the first requirement to perform Spoligotyping based molecular investigation, the current manuscript was done from few selected samples.

Indeed, we acknowledge the comments and already stated in the limitation section as “**the fairly small sample size, uneven representation of strains from the study sites, and further categorization into different levels of factor variables, has reduced the power of our statistical analysis**.” Not only this, there is detailed explanation on the study limitation that we forwarded a message to readers that the numerical estimates may not truly imitate the situation for the general population and need to cautions in the interpretation of findings. Indeed, as much as possible we tried to minimize most of those methodological limitation (anticipated bias) using advanced statistical methods.
